# Supplementary material for: AI Pontryagin or how artificial neural networks learn to control dynamical systems
Source: Nat Commun. 2022 Jan 17;13:333. doi: 10.1038/s41467-021-27590-0 (PMC8763915; doi:10.1038/s41467-021-27590-0)
Supplement: Supplementary file 1 — Supplemental Information [file 41467_2021_27590_MOESM1_ESM.pdf]

SUPPLEMENTAL INFORMATION
AI Pontryagin or How Artificial Neural Networks Learn to Control Dynamical Systems

Lucas Böttcher,[1,2,*] Nino Antulov-Fantulin,[3,†] and Thomas Asikis[3,‡]

[1] *Computational Social Science, Frankfurt School of Finance and Management, Frankfurt am Main, 60322, Germany*

[2] *Dept. of Computational Medicine, University of California, Los Angeles, 90095-1766, Los Angeles, United States*

[3] *Computational Social Science, ETH Zurich, 8092, Zurich, Switzerland*

(Dated: November 21, 2021)

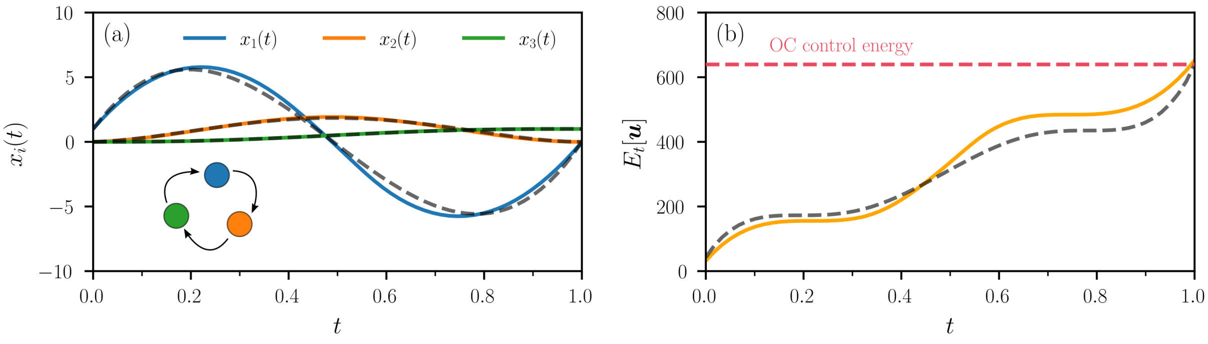

FIG. S1. **Controlling a connected 3-node cogwheel system.** **(a)** Evolution of system states $x_i(t)$ $(1 \leq i \leq 3)$ under OC (dashed lines) and neural-network controls (solid lines). **(b)** Evolution of the control energy $E_t[\boldsymbol{u}]$ for AI Pontryagin after 20,000 training epochs (solid orange line) and optimal control (dashed black line). The learning rate is $\eta = 6 \times 10^{-4}$.

I. DIRECTED NETWORKS

This section discusses three different examples of directed networks which we control with AI Pontryagin.

As a first example, we study a 3-node cogwheel system with adjacency and driver matrices

$$A = \begin{pmatrix} 0 & 0 & 1 \\ 1 & 0 & 0 \\ 0 & 1 & 0 \end{pmatrix} \quad \text{and} \quad B = \begin{pmatrix} 1 \\ 0 \\ 0 \end{pmatrix} . \tag{S1}$$

Observe that the circulant matrix A satisfies $A^3 = \mathbb{1}$, so the spectrum of A is $\lambda(A) = \{1, e^{-2\pi \mathrm{i}/3}, e^{2\pi \mathrm{i}/3}\}$. The same holds for the transpose $A^\top$. Since A, $A^\top$ are diagonalizable, we can directly evaluate the corresponding matrix exponentials in the controllability Gramian $W(t)$. Figure S1 shows the evolution of $x_i(t)$ $(1 \leq i \leq 3)$ for the system that is subject to optimal control (OC) and AI Pontryagin-based control signals. The initial and target states are $\boldsymbol{x}(0) = (1, 0, 0)^\top$ and $\boldsymbol{x}^* = (0, 0, 1)^\top$, respectively. We observe that both control approaches reach the desired target state for $T = 1$. The control energy associated with AI Pontryagin also resembles that of OC. The relative difference at time $T = 1$ is about 2%.

* l.boettcher@fs.de
† anino@ethz.ch
‡ asikist@ethz.ch

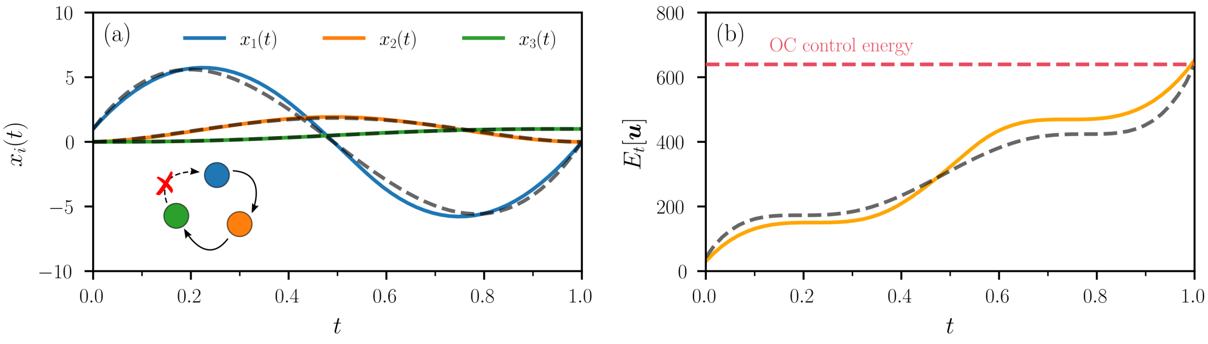

FIG. S2. **Controlling an unconnected 3-node cogwheel system.** **(a)** Evolution of system states $x_i(t)$ $(1 \leq i \leq 3)$ under OC (dashed lines) and neural-network controls (solid lines). **(b)** Evolution of the control energy $E_t[\boldsymbol{u}]$ for AI Pontryagin after 20,000 training epochs (solid orange line) and optimal control (dashed black line). The learning rate is $\eta = 6 \times 10^{-4}$.

The artificial neural network (ANN) that we use to control the 3-node system consists of a single hidden layer with 20 neurons and an exponential linear unit (ELU) activation. We transform the hidden layer output to a control signal via a linear layer with 1 neuron that describes the single control input. We initialize the ANN weights $\boldsymbol{w}$ with the Kaiming uniform initialization algorithm [1]. For the gradient descent in $\boldsymbol{w}$ [Eq. (4) in the main text], we use a learning rate $\eta = 6 \times 10^{-4}$. The number of time steps is 60.

As a second example, we consider the following directed system with an absorbing state and a non-invertible adjacency matrix

$$A = \begin{pmatrix} 0 & 0 & 0 \\ 1 & 0 & 0 \\ 0 & 1 & 0 \end{pmatrix} \quad \text{and} \quad B = \begin{pmatrix} 1 \\ 0 \\ 0 \end{pmatrix} . \tag{S2}$$

Observe that A and $A^\top$ are nilpotent matrices of degree 3. The matrix exponentials arising in the controllability Gramian can be directly calculated from their series expansions since the series have non-zero terms only for A^k with $k < 3$. For the same initial and target states as in the previous example, we show the evolution of $x_i(t)$ $(1 \leq i \leq 3)$ and $E_t[\boldsymbol{u}]$ in Fig. S2.

In the third example, we focus on a directed growing network [2] with 1,024 nodes. We select different proportions of controlled nodes uniformly at random. If 95% of all nodes are

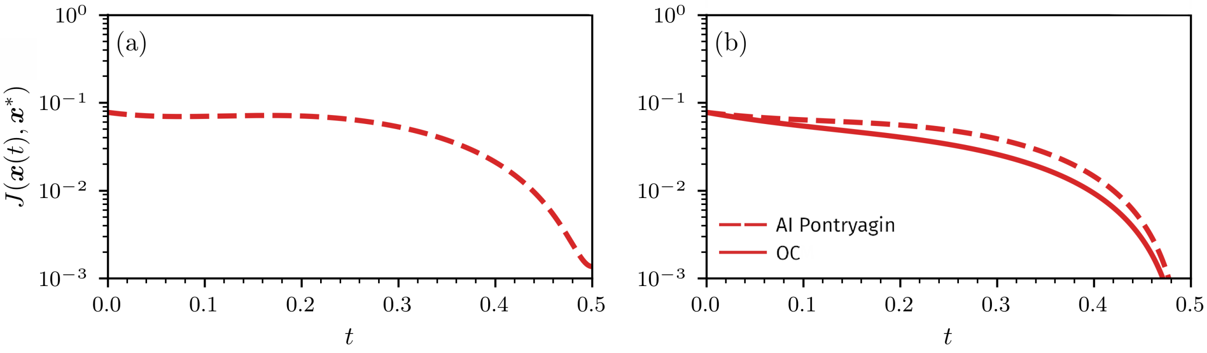

FIG. S3. **Controlling a larger-scale directed network.** We use AI Pontryagin to control a growing network [2] (directed graph) with 1,024 nodes for two different fractions of controlled nodes that are selected uniformly at random. **(a)** If 95% of all nodes are controlled, the controllability Gramian in Eq. (7) (see main text) is non-invertible and OC solutions (6) (see main text) cannot be calculated. AI Pontryagin still manages to steer the dynamics towards the target state and minimize the final loss $J(\boldsymbol{x}(T), \boldsymbol{x}^*)$. **(b)** For 99% controlled nodes, both methods minimize the loss function. Dashed and solid lines indicate AI Pontryagin and OC solutions, respectively.

controlled, OC solutions cannot be calculated analytically since the controllability Gramian $W(T)$ [Eq. (7) in the main text] is not invertible, implying that the linear dynamical system is not controllable. For larger proportions of controlled nodes, the OC approach gains in its ability to control the directed network. AI Pontryagin is able to minimize the loss function and steer the system very close to the desired target state for both examples shown in Fig. S3. The relative difference between the control energies of AI Pontryagin and OC for the solution shown in Fig. S3 is about 20%. In this example, node states are initialized with values uniformly sampled from the interval $[0, 1]$. The target state is generated by applying update rule (S4) to the initial state for 40 iterations. The artificial neural network that we use to control the directed network consists of a single hidden layer with 15 ELUs. We used a learning rate $\eta = 1.2$.

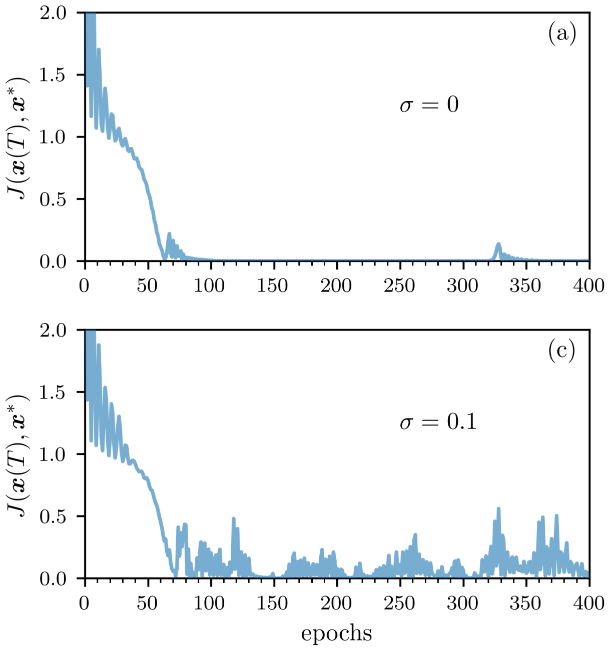

FIG. S4. **Effect of noise on learning performance of AI Pontryagin with fixed learning rate.** For the two-state system (8) (see main text) and different noise levels [$\sigma = 0$ in **(a)**, $\sigma = 0.05$ in **(b)**, $\sigma = 0.1$ in **(c)** and $\sigma = 0.5$ in **(d)**], we show the loss (5) (see main text) as a function of the number of training epochs. Gaussian noise with zero mean and variance σ^2 acts on the observed reached state $\boldsymbol{x}(T)$ according to Eq. (S3). We set the learning rate to a value of $\eta = 0.1$ and use the Adam optimizer.

II. NOISE ROBUSTNESS

To study the robustness of the backpropagation of loss function gradients during training, we carry out additional numerical experiments with additive noise that acts as uncertainty either on the observed reached state or on the target state at time T. That is,

$$\hat{\boldsymbol{x}}(T) = \boldsymbol{x}(T) + \boldsymbol{\epsilon} \quad \text{or} \quad \hat{\boldsymbol{x}}^* = \boldsymbol{x}^* + \boldsymbol{\epsilon}\,, \tag{S3}$$

where $\boldsymbol{x}(T)$ and $\boldsymbol{x}^*$ respectively denote the unperturbed reached state and unperturbed target state, and $\boldsymbol{\epsilon}$ is a vector whose elements are distributed according to a Gaussian $\mathcal{N}(0, \sigma)$ with

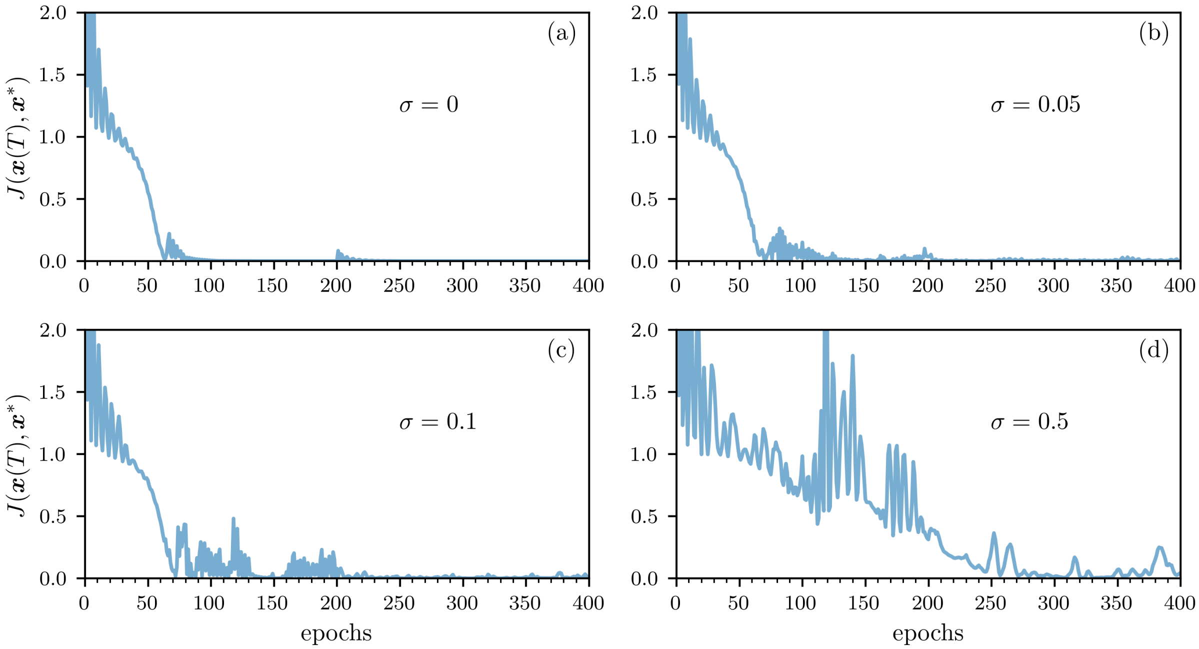

FIG. S5. **Effect of noise and adaptive learning rates on learning performance of AI Pontryagin.** For the two-state system (8) (see main text) and different noise levels [$\sigma = 0$ in **(a)**, $\sigma = 0.05$ in **(b)**, $\sigma = 0.1$ in **(c)** and $\sigma = 0.5$ in **(d)**], we show the loss (5) (see main text) as a function of the number of training epochs. Gaussian noise with zero mean and variance σ^2 acts on the observed reached state $\boldsymbol{x}(T)$ according to Eq. (S3). We initially set the learning rate to a value of $\eta = 0.1$. After 200 training epochs, we set $\eta = 0.01$ as a fine-tuning mechanism. Computations were performed with the Adam optimizer.

zero mean and variance σ^2. The uncertainty associated with the observed reached state acts as a perturbation on the loss function and its gradients. If the signal to noise ratio is not too large, gradients still carry enough information for efficient learning of control signals (see Fig. S4). The level of observation-noise fluctuations (modeled by σ) and the gradient-descent learning rate η both affect the convergence of the learning procedure. By adaptively changing the learning rate, we reduce the impact of noise on convergence (see Fig. S5). To improve noise robustness of the training process, one can use different learning rate schedulers[1] in `PyTorch`. A more in-depth analysis of the interplay of noise, learning rates, and stiffness of controlled differential equations is an interesting direction for future work.

[1] `https://pytorch.org/docs/stable/optim.html`

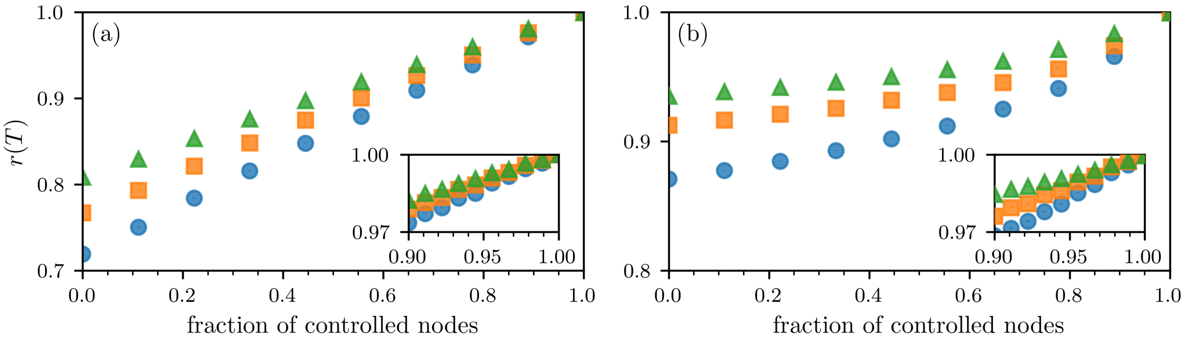

FIG. S6. **Dependence of the order parameter on the fraction of driver nodes.** The order parameter $r(T)$ [Eq. (20) in the main text] of Kuramoto oscillator dynamics as a function of the fraction of controlled nodes for **(a)** an Erdős–Rényi network $G(N, p)$ with $p = 0.3$ and **(b)** a Watts–Strogatz network with degree $k = 5$ and rewiring probability $p = 0.3$. Both graphs have $N = 225$ nodes. Hence, the mean degree of the Erdős–Rényi network is 67.5. We set $T = 5$ and used learning rates η between 0.1 and 1.0. The shown results are based on averages over 1,000 i.i.d. natural frequency realizations. Different markers indicate different coupling constants: $K = 0.1K^*$ (blue disks), $K = 0.15K^*$ (orange squares), and $K = 0.2K^*$ (green triangles).

III. DRIVER NODES

A. Kuramoto dynamics

To study the influence of different fractions of controlled (*i.e.*, driver) nodes on the control performance of AI Pontryagin, we consider an Erdős–Rényi and a Watts–Strogatz network with $N = 225$ nodes (see Fig. 4 in the main text) and different numbers of uniformly at random selected driver nodes. Neural-network architectures are as reported in the main text.

Figure S6 shows the order parameter $r(T)$ [Eq. (20) in the main text] for $T = 5$ and different driver node fractions. The coupling constant K is set to 10, 15, and 20% of the critical coupling constant K^*. For both networks, we observe that the order parameter is between 0.97 and 1 for driver-node fractions larger than 90%. For a fraction of controlled nodes of 95%, the order parameters are 0.987 ($K = 0.1K^*$), 0.990 ($K = 0.15K^*$), and 0.992

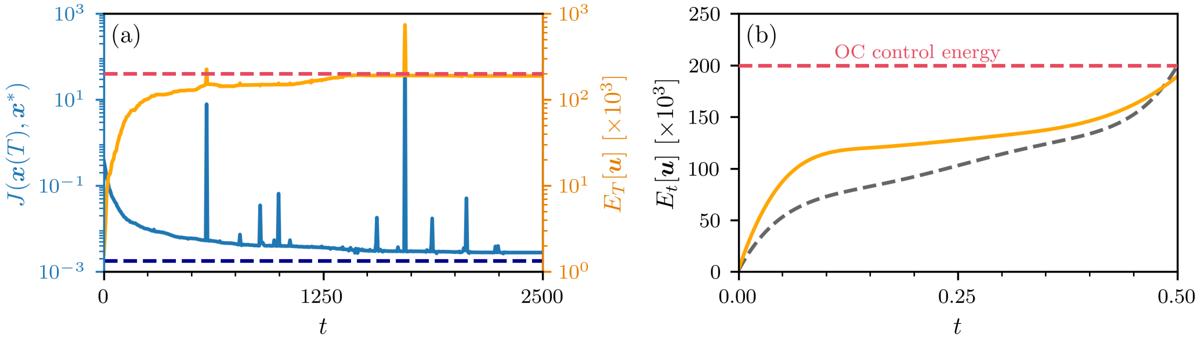

FIG. S7. **Control of linear systems with a reduced set of controlled nodes. (a)** Final loss [Eq. (5) in the main text] and control energy [Eq. (3) in the main text] as a function of training epochs. Dashed and solid lines indicate OC and AI Pontryagin-based solutions. **(b)** Total control energy of AI Pontryagin (solid orange line) and OC (dashed grey line). The optimal control solution is based on Eq. (6) in the main text. The fraction of controlled nodes in (a,b) is 50%. We determined driver matrix B and driver nodes according to the maximum matching method [4].

($K = 0.2K^*$) in the Erdős–Rényi network, and 0.983 ($K = 0.1K^*$), 0.988 ($K = 0.15K^*$), and 0.992 ($K = 0.2K^*$) in the Watts–Strogatz network. For smaller fractions of controlled nodes, Fig. S6 shows that the order parameter in the Erdős–Rényi network is more sensitive to the fraction of controlled nodes than in the Watts–Strogatz network. These findings are well-aligned with those reported in [3], indicating that in oscillator networks with a large mean degree and a small coupling constant, a large proportion of nodes need to be controlled to achieve a certain degree of synchronization. The order parameter in networks with smaller mean degrees and a subcritical coupling constant is less sensitive to the fraction of controlled nodes.

B. Linear dynamics

Next, we discuss how linear systems can be controlled with a subset of all nodes (*i.e.*, driver nodes). We set A in $f(\boldsymbol{x}, \boldsymbol{u}) = A\boldsymbol{x} + B\boldsymbol{u}$ (see main text) equal to the adjacency matrix of a lattice graph with $N = 1,024$ nodes. To determine the driver matrix B and driver nodes, we use the maximum matching method [4]. The corresponding fraction of driver nodes is 50%

for the considered lattice graph. In our numerical experiments, node states are initialized with values uniformly sampled from the interval $[-1, 1]$. The target states $\boldsymbol{x}^*$ are chosen according to a deterministic cellular automaton rule that produces a pattern in which node states are correlated with the states of their neighbors. The update rule is

$$x_i^{*(n+1)} = 0.95 x_i^{*(n)} + 0.05 \max_{j \in N_i} |A_{ij} x_j^{*(n)}| \,, \tag{S4}$$

where N_i denotes the neighborhood of node i. For the target state that we use in our simulations, we perform the above iteration for a number of times which is uniformly sampled from the set $\{50, 100, \ldots, 500\}$. For a large number of iterations, the update rule (S4) creates clusters in which nodes share similar state values. Ultimately, all node states will converge to the initial state value with the highest absolute value.

Figure S7 shows that AI Pontryagin is able to reach loss and control energy values that are similar to those of optimal control. The neural network that we use in this simulation has 1 hidden layer with 15 ELU hidden units. Bias terms are included as inputs in all layers. Further details about the training process are publicly available at [5].

IV. REGULARIZATION PARAMETER OF THE ADJOINT GRADIENT METHOD

The control energy term in the adjoint-gradient method (AGM) depends on the regularization parameter β. In Fig. S8, we compare the performance of the AGM and AI Pontryagin to control coupled Kuramoto oscillators on a square lattice with $N = 225$ nodes for different AGM regularization parameters β ranging from 10^{-7} to 10^{-1}. If the regularization parameter β is too large, the energy term and not the cost associated with a low degree of synchronization dominates, and the AGM fails to synchronize the oscillator system, leading to small order parameters and large control energies.

AI Pontryagin has no explicit energy regularization hyperparameter in the sense of β. It relies on an implicit energy regularization, resulting from the interplay of ANN initialization and an induced gradient descent (see main text). For a more detailed comparison with the AGM, we analyze the dependence of both the order parameter and control energy on different

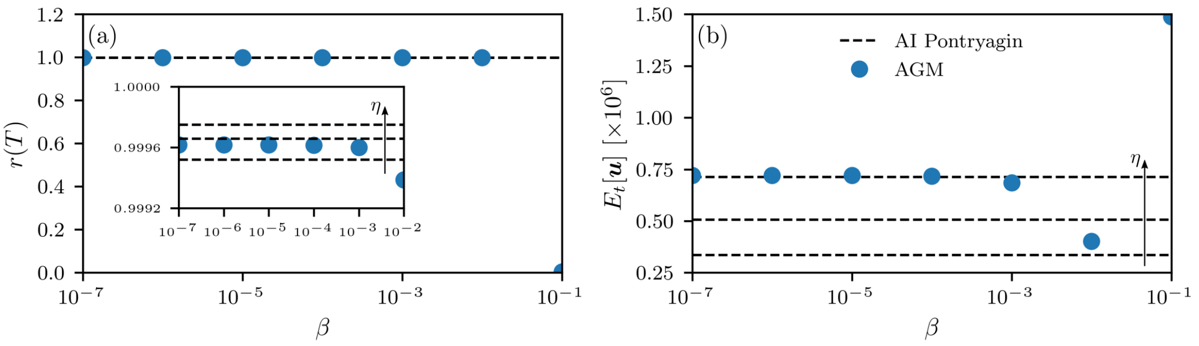

FIG. S8. **Controlling coupled oscillators with AI Pontryagin and the AGM.** We test the performance of AI Pontryagin and AGM [Eqs. (17) and (18) in the main text] to control coupled Kuramoto oscillators on a square lattice with $N = 225$ nodes and different AGM regularization parameters β ranging from 10^{-7} to 10^{-1}. The total simulation time is $T = 3$. **(a)** The order parameter $r(T)$. **(b)** The control energy $E_t[\boldsymbol{u}]$. Dashed horizontal lines indicate AI Pontryagin solutions (with different learning rates η= 0.22,0.27,0.32) and blue disks indicate AGM solutions ($\tilde{\eta} = 25$).

AI Pontryagin learning rates η. We observe that AI Pontryagin achieves stable control of the oscillator system, as indicated by the order parameter value of $r(T) \approx 1$. At the same time, different learning rates may lead to different control energies $E_t[\boldsymbol{u}]$. Small learning rates allow AI Pontryagin to explore the dynamical system in more detail, leading to smaller control energies and nearly optimal control solutions.

V. RUNTIME COMPARISON

In this section, we provide a more detailed comparison of the runtime complexity of AI Pontryagin and the AGM. To do so, we study the performance of both methods in controlling systems of Kuramoto oscillators whose connections are described by Erdős–Rényi networks. As in the main text, we use a subcritical coupling constant $K = 0.1K^*$ and compare trajectories for which order parameter and control energy values of both methods are similar. Learning rates are as reported in Tab. I.

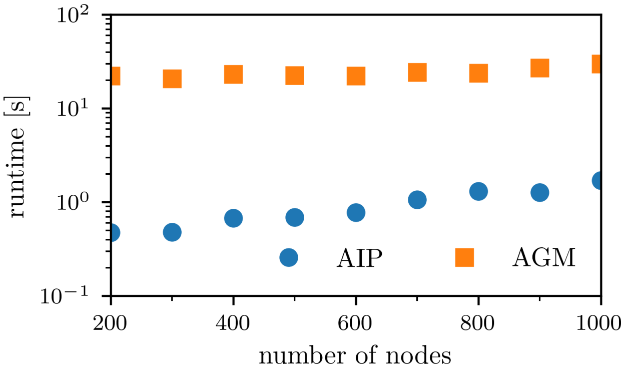

FIG. S9. **Runtime comparison between AI Pontryagin and the adjoint-gradient method.** We show the runtime needed to control a network of coupled Kuramoto oscillators with a subcritical coupling constant $K = 0.1K^*$. Blue disks indicate the runtime of AI Pontryagin (AIP) and orange squares indicate the runtime of the adjoint-gradient method (AGM). Simulations were performed on an Erdős–Rényi network $G(N, p)$ with $p = 0.3$. The number of time steps is 60 and the number of oscillators N ranges from 200 to 1,000. Reported runtimes are averaged over 10 realizations. Error bars are smaller than the markers.

Figure S9 shows a runtime comparison for 9 different system sizes. We observe that the mean runtime is between 0.5 and 1.5 seconds for AI Pontryagin and it is between 20 and 30 seconds for the AGM. What are the main features in the respective control architectures that are associated with these remarkable 20 to 30-fold speedups of AI Pontryagin? To numerically solve the underlying dynamical systems, we use the Dormand–Prince (DOPRI) method with adaptive step size [6] in both control frameworks. For a network with N nodes, the AGM relies on the numerical integration of $2N$ nonlinear differential equations, describing the coupled primal and adjoint equations [Eqs. (11), (12), and (18) in the main text], at every iteration step. After solving the primal and adjoint systems, the AGM calculates a new estimate of the optimal control function according to the gradient descent (17) (see main text). To solve control problems with AI Pontryagin, one needs to numerically integrate the primal system (not the adjoint system) and backpropagate gradients. Differences in the runtime performance between both control frameworks are associated with stiffness problems that may arise in the numerical solution of the coupled primal and adjoint equations [Eqs. (11), (12), and (18) in the main text], and the corresponding gradient descent in the control

function [Eq. (17) in the main text]. To identify the main computational bottlenecks in the AGM, we performed a detailed runtime analysis of all code segments and found that the adjoint system solver requires very small step sizes to resolve the interaction between the adjoint system and the gradient descent in the control functions. One possibility to further improve the performance of the AGM is to use a problem-tailored initializations of the control function. Such an approach requires knowledge on the mathematical structure of the optimal control signal which may not be possible to obtain, in particular for high-dimensional and analytically intractable control problems. In our simulations, we use a uniform random initialization with support $[1, 2]$ to align the runtimes with those reported in [7]. We also tested other initialization protocols (*e.g.*, $u^{(0)}(t) = 1$ and $u^{(0)}(t) = t/T$) and were able to reduce the runtime for an Erdős–Rényi network with 225 nodes by a factor between 2–3. Still, for this example, AI Pontryagin is about 5–10 times faster, without optimizing the initial guess $u^{(0)}(t)$.

Edges (undirected)	Nodes	Learning rate η	Learning rate $\tilde{\eta}$
5,968	200	0.05	0.6
13,464	300	0.05	0.6
23,865	400	0.05	0.6
37,422	500	0.05	0.6
53,897	600	0.05	0.65
73,444	700	0.05	0.65
96,034	800	0.05	0.6
121,320	900	0.05	0.6
149,938	1,000	0.05	0.6

TABLE I. Learning rates η (AI Pontryagin) and $\tilde{\eta}$ (AGM) used for learning the control of Kuramoto dynamics on Erdős–Rényi networks with different numbers of nodes. All ANNs use stochastic gradient descent for learning and only differ in their learning rate. The number of hidden layers and hidden layer neurons are 1 and 2, respectively. We use an ELU activation function and train the ANN for three epochs. At each node, we include a bias term and set all weights initially to a value of 10^{-3}. The energy regularization parameter of the AGM is set to $\beta = 10^{-7}$.

[1] K. He, X. Zhang, S. Ren, and J. Sun, in *Proc. IEEE Int. Conf. Comput. Vis.* (2015) pp. 1026–1034.

[2] P. L. Krapivsky and S. Redner, Phys. Rev. E **63**, 066123 (2001).

[3] P. S. Skardal and A. Arenas, Sci. Adv. **1**, e1500339 (2015).

[4] Y.-Y. Liu, J.-J. Slotine, and A.-L. Barabási, Nature **473**, 167 (2011).

[5] "Code and data repository, `https://github.com/asikist/nnc`,".

[6] J. R. Dormand and P. J. Prince, J. Comput. Appl. **6**, 19 (1980).

[7] U. Biccari and E. Zuazua, Front. Energy Res. **8** (2020).
